# Crystal structure and Hirshfeld surface analysis of 2-(2-hy­droxy­phen­yl)quinoline-6-sulfonamide

**DOI:** 10.1107/S2056989022002870

**Published:** 2022-03-17

**Authors:** Nesrine Benarous, Nabila Moussa Slimane, Hassiba Bougueria, Mehdi Boutebdja, Aouatef Cherouana

**Affiliations:** a Unité de Recherche de Chimie de l’Environnement et Moléculaire Structurale (CHEMS), Faculté des Sciences Exactes, Université Frères Mentouri Constantine 1, Constantine, 25017, Algeria; b Centre Universitaire Abd El Hafid Boussouf, Mila, 43000 Mila, Algeria; c Laboratoire de Technologie des Matériaux Avancés, École Nationale Polytechnique de Constantine, Nouvelle Ville Universitaire, Ali Mendjeli, Constantine 25000, Algeria

**Keywords:** quinoline derivatives, single-crystal X-ray diffraction, hydrogen bonding, inter­molecular inter­actions, Hirshfeld surface analysis

## Abstract

In the title compound, C_15_H_12_N_2_O_3_S, there are two mol­ecules (*A* and *B*) in the asymmetric unit. The attached phenol and quinoline moieties of each mol­ecule are almost coplanar with a dihedral angle of 6.05 (15)° for mol­ecule *A* and 1.89 (13)° for mol­ecule *B*. The crystal structure features N—H⋯O and C—H⋯O hydrogen bonds, C—H⋯π inter­actions and π–π stacking inter­actions. Hirshfeld surface analysis indicates that the most significant contacts in the crystal packing are C⋯H/H⋯C (29.2%), O⋯H/H⋯O (28.6%) and H⋯H (28.5%).

## Abstract 2

The asymmetric unit of 2-(2-hy­droxy­phen­yl)quinoline-6-sulfonamide contains two crystallographically independent mol­ecules. The crystal structure features hydrogen bonding and π–π stacking inter­actions.

## Chemical context

Quinolines are well-known heterocyclic compounds and have been used successfully in many pharmacological and medicinal fields, exhibiting biological properties including anti­cancer, anti­malarial, anti­bacterial, anti­asthmatic and antihypertensive activities (Chi *et al.*, 2018 ▸; Ferreira *et al.*, 2020 ▸; Elgawad *et al.*, 2019 ▸; Mulakayala *et al.*, 2012 ▸; Lavanya *et al.*, 2021 ▸; Yadav & Shah, 2021 ▸; Shishkina *et al.*, 2018 ▸). In addition, quinolines and/or their metal complexes have a wide range of physical and chemical applications. They have been used in fields such as coordination chemistry (Twaróg *et al.*, 2020 ▸), metal–organic frameworks (MOFs) (Wu *et al.*, 2015 ▸), catalysis (Redshaw & Tang, 2012 ▸), textile printing (Hassan *et al.*, 2022 ▸), food additives (Al-Shabib *et al.*, 2020 ▸), anti-corrosion (Galai *et al.*, 2021 ▸), photoluminescence (Twaróg *et al.*, 2020 ▸), magnetism (Yu *et al.*, 2019 ▸) and non-linear optics (Goel *et al.*, 2018 ▸). 
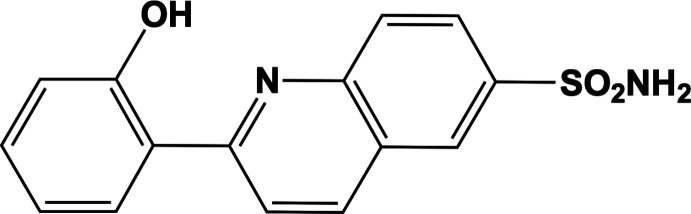


We report here the synthesis, structural characterization and Hirshfeld surface analysis of a new quinoline derivative, 2-(2-hy­droxy­phen­yl)quinoline-6-sulfonamide. This compound was prepared in a two-step reaction, *viz.* reflux and solvothermal (see *Synthesis and crystallization* section).

## Structural commentary

**Figure 1:**
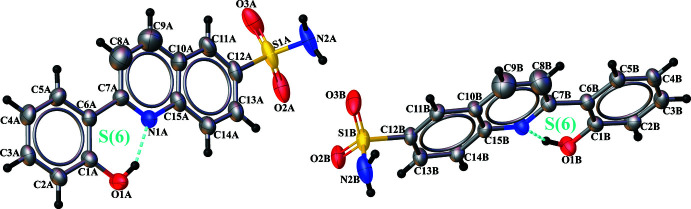
View of the two independent mol­ecules of the title compound, showing the atom-labeling scheme. Displacement ellipsoids are drawn at the 50% probability level. Intra­molecular hydrogen bonds are shown as dashed cyan lines.

The asymmetric unit of title compound (I), illustrated in Fig. 1 ▸, contains two crystallographically independent mol­ecules (*A* and *B*). The C6*A*—C7*A* and C6*B*—C7*B* bond lengths of 1.472 (5) and 1.470 (5) Å, respectively, are notably shorter than the normal C—C single bond due to conjugation but are comparable to those observed in related structures (Shrungesh Kumar *et al.*, 2015 ▸; Mague *et al.*, 2016 ▸).

**Figure 2:**
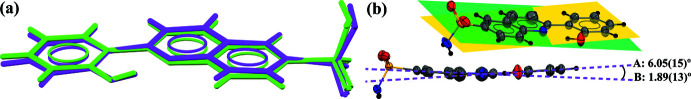
(*a*) Overlay image of the two mol­ecules in the asymmetric unit of the title compound. (*b*) Dihedral angles between the quinoline and the phenol moieties in the title compound.

The hydroxyl group in the *ortho*-position of each independent mol­ecule in (I) allows the formation of an intra­molecular O—H⋯N hydrogen bond, generating an *S*(6) ring motifs (Fig. 1 ▸, Table 1 ▸), which stabilize the mol­ecules and also affect the overall mol­ecular conformation. The conformational differences between mol­ecules *A* and *B* are highlighted in an overlay diagram shown in Fig. 2 ▸ *a*. The two rings of the quinoline system are fused almost coaxially (r.m.s. deviation = 0.004 Å), with a dihedral angle between their planes of 4.0 (2)° for mol­ecule *A* and 1.49 (17)° for mol­ecule *B*.

The attached quinoline and phenol moieties are almost coplanar with a dihedral angle of 6.05 (15)° for mol­ecule *A* and 1.89 (13)° for mol­ecule *B* (Fig. 2 ▸ *b*), indicating a significant electron delocalization within the mol­ecules. The sulfonamide groups are twisted away from the attached quinoline fragment with an C11*A*—C12*A*—S1*A*—N2*A* torsion angle of 91.8 (4)° for mol­ecule *A* and C11*B*—C12*B*—S1*B*—N2*B* torsion angle of − 79.9 (3)° for mol­ecule *B*. The sulfonamide atoms S1*A* and S1*B* deviate by 0.228 (1) and 0.054 (1) Å from the planes of the quinoline fragment in mol­ecules *A* and *B* respectively.

## Supra­molecular features

**Figure 3:**
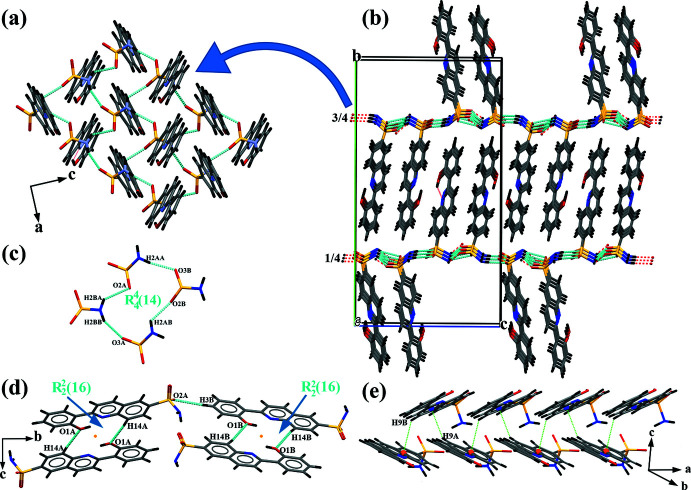
Part of the crystal structure of the compound (I) showing (*a*) a view along the *b* axis of the two-dimensional hydrogen-bonded network; (*b*) the two-dimensional network parallel to the *ac* plane at 1/4 and 3/4 of the *b-*axis length; (*c*) the N—H⋯O hydrogen bonds of the sulfonamide groups generating an 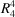 (14) motif; (*d*) C—H⋯O hydrogen bonds generating an inversion dimer with an 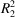 (16) ring motif and (*e*) the C—H⋯π inter­action generating a chain running along the *a-*axis direction.

In the crystal of (I), the presence of sulfonamide group leads indeed to the formation of strong inter­molecular N—H⋯O hydrogen bonds (Table 1 ▸), generating supra­molecular hydrogen-bonded layers parallel to the (010) plane (Fig. 3 ▸ *a*). The packing diagram of the title compound viewed down the *a* axis (Fig. 3 ▸ *b*) shows that the layers are stacked perpendicular to the *b* axis at (0,1/4,0) and (0,3/4,0). These layers are formed by aggregation of 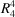 (14) ring motifs (Fig. 3 ▸ *c*). In addition, the hydroxyl group of each mol­ecule is involved in a C—H⋯O hydrogen bond, forming an inversion dimer with an 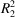 (16) graph-set motif. The dimers are linked by a further C—H⋯O hydrogen bond involving one of the oxygen atoms of the sulfonamide group (Fig. 3 ▸ *d*). Weak inter­molecular C—H⋯π inter­actions are also observed in the crystal packing, forming a chain along the *a*-axis direction (Fig. 3 ▸ *e*).

**Figure 4:**
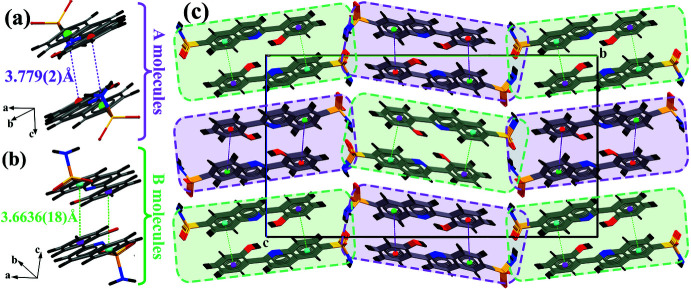
π–π stacking inter­actions in (I), showing (*a*) the resulting stacks formed by the *A* mol­ecules; (*b*) a similar view showing the stacks formed by the *B* mol­ecules and (*c*) a view along the *a* axis of the stacked *A* and *B* mol­ecules. Dashed magenta lines denote *Cg*2⋯*Cg*3 contacts and dashed light-green lines *Cg*6⋯*Cg*7 contacts.

Cohesion of the crystal structure is enhanced by the presence of π–π stacking inter­actions, the most significant being between the 2-hy­droxy­phenyl and benzene rings of the quinoline groups of each mol­ecule [*Cg*2⋯*Cg*3(−*x*, −*y*, 1 − *z*) = 3.779 (2) Å (Fig. 4 ▸ *a*) for *A* mol­ecules and *Cg*6⋯*Cg*7(1 − *x*, 1 − *y*, 1 − *z*) = 3.6636 (18) Å (Fig. 4 ▸ *b*) for *B* mol­ecules where *Cg*2, *Cg*3, *Cg*6 and *Cg*7 are the centroids of the C1*A*–C6*A*, C10*A*–C15*A*, C1*B*–C6*B* and C10*B*–C15*B* rings, respectively]. These result in the formation of a supra­molecular ribbon parallel to the *a* axis based on the stacked mol­ecules (Fig. 4 ▸ *c*).

## Hirshfeld surface analysis

**Figure 5:**
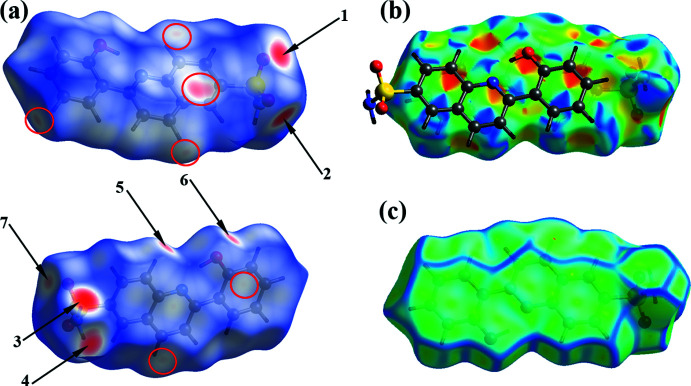
A view of the Hirshfeld surface for (I) mapped over (*a*) *d*_norm_ in the range −0.5231 to +1.1263 arbitrary units, (*b*) shape-index and (*c*) curvedness.

For further characterization of the inter­molecular inter­actions in (I), we carried out a Hirshfeld surface (HS) analysis (Spackman & Jayatilaka, 2009 ▸) using *CrystalExplorer* (Spackman *et al.*, 2021 ▸) and generated the associated two-dimensional fingerprint plots (McKinnon *et al.*, 2007 ▸). The HS of (I) mapped over *d*_norm_ in the range −0.5231 to +1.1263 a.u. is illustrated in Fig. 5 ▸ *a* using color to indicate contacts that are shorter (red areas), equal to (white areas), or longer than (blue areas) the sum of the van der Waals radii. The dominant inter­actions between sulfonamide N—H and O atoms can be seen as the bright-red areas marked as 1, 2, 3 and 4. The light-red spots labeled as 5, 6 and 7 are due to C—H⋯O inter­actions. The weak C—H⋯π contacts are indicated by the red ellipse.

The presence of characteristic triangles on the shape-index surface (Fig. 5 ▸ *b*) clearly indicate the presence of π–π inter­actions between neighboring mol­ecules while the curvedness plots (Fig. 5 ▸ *c*) show flat surface patches characteristic of planar stacking.

**Figure 6:**
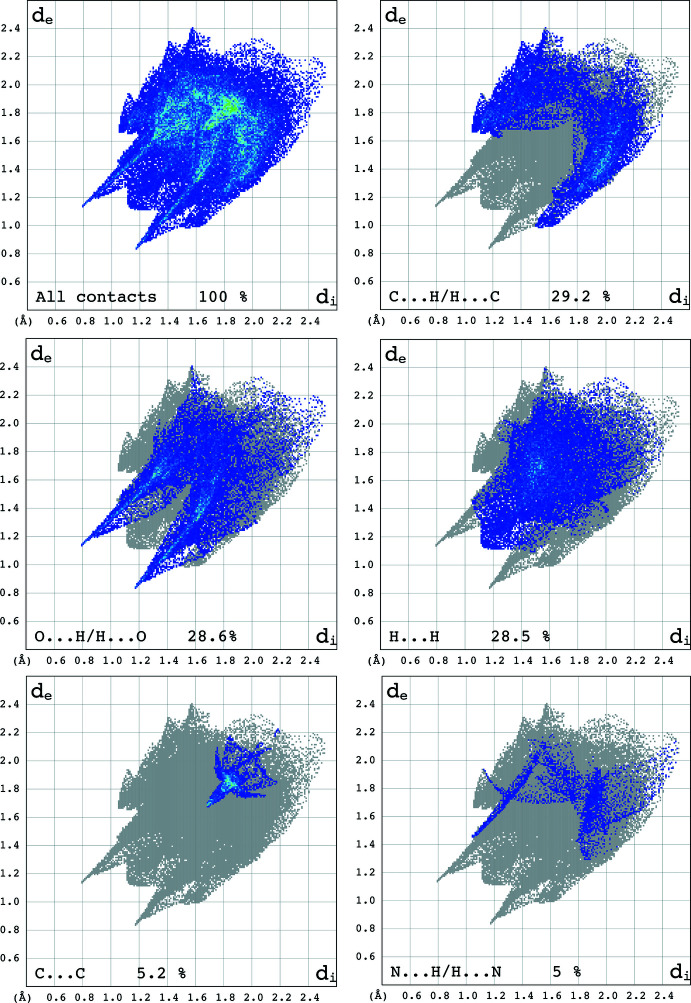
Two-dimensional fingerprint plots for (I), showing the contributions of all contacts and and those delineated into C⋯H/H⋯C, O⋯H/H⋯O, H⋯H, C⋯C and N⋯H/H⋯N contacts.

**Figure 7:**
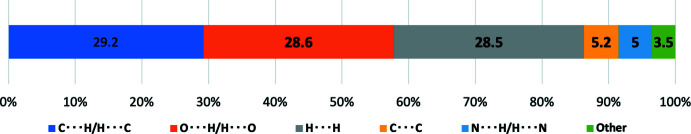
Percentage contributions of contacts to the Hirshfeld surface in the title compound.

**Figure 8:**
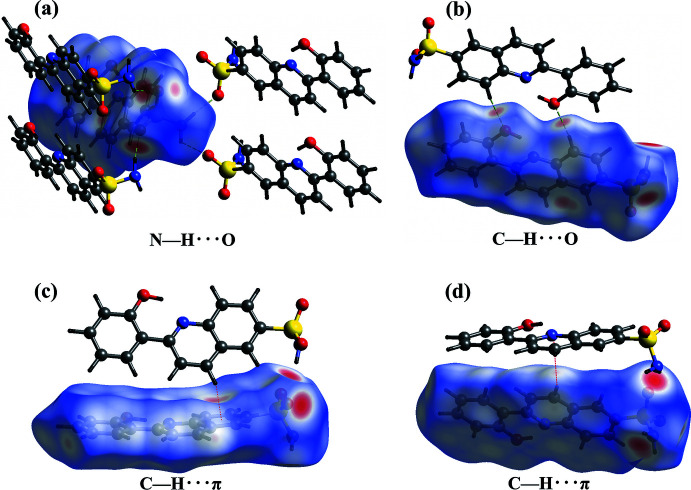
Views of the Hirshfeld surface mapped over *d*_norm_ showing (*a*) and (*b*) O⋯H/H⋯O contacts, and (*c*) and (*d*) C⋯H/H⋯C contacts.

The overall two-dimensional fingerprint plot and those delineated into C⋯H/H⋯C, O⋯H/H⋯O, H⋯H, C⋯C and N⋯H/H⋯N contacts are illustrated in Fig. 6 ▸ together with their relative contributions to the Hirshfeld surface. The fingerprint plots show that the C⋯H/H⋯C contacts (29.2%) make the largest contribution to the overall packing of the crystal (Table 2 ▸, Fig. 7 ▸), which are related to the presence of C—H⋯π inter­actions in the structure of (I) (Fig. 8 ▸ *c*–*d*).

The second most important inter­actions are O⋯H/H⋯O contributing by 28.6% to the overall crystal packing (Table 2 ▸, Fig. 6 ▸), and are related to the presence of N—H⋯O and C—H⋯O inter­actions in the structure of (I) (Fig. 8 ▸ *a*,*b*). In addition, van der Waals inter­actions (H⋯H) are one of the major (28.5%) inter­actions in this structure. The presence of weak π–π stacking inter­actions are reflected in the 5.2 and 1.2% contributions from C⋯C and C⋯N/N⋯C contacts to the Hirshfeld surface. Other contacts make a contribution of 3.5% in total and are not discussed in this work.

## Database survey

A search for 2-hy­droxy­phenyl­quinoline in the Cambridge Structural Database (CSD; Version 2021.3.0, last update November 2021; Groom *et al.*, 2016 ▸) gave 29 hits, which exhibit structural diversity with inter­esting properties, such as chemical (Alexandre *et al.*, 2020 ▸; Han *et al.*, 2017 ▸; Yao *et al.*, 2012 ▸; Guo *et al.*, 2006 ▸), physical (Zheng *et al.*, 2013 ▸; Elbert *et al.*, 2017 ▸) and biological (Mulakayala *et al.*, 2012 ▸).

## Synthesis and crystallization

The title compound was prepared by a two-step reaction. First, an ethanol solution (5 mL) of 4-amino­benzene­sulfonamide (0.33 g, 1.9 mmol) was added dropwise under stirring to an ethanol solution (5 mL) of 2-hy­droxy­benzaldehyde (0.2 mL, 0.234 g, 1.9 mmol) and refluxed for 2 h. After that, an acetone solution (5 mL) of palladium(II) acetate (0.05 g, 0.2 mmol) was added dropwise under stirring for 1 h. The yellow mixture was then transferred to a 25 mL Teflon-lined stainless-steel autoclave and sealed to heat at 393 K. After reaction for 48 h, the autoclave was cooled down to room temperature. Yellow block-like crystals suitable for X-ray diffraction analysis were obtained, isolated by filtration, washed with water and dried in air. Yield: 0.25 g, 43.44%.

## Refinement

Crystal data, details of data collection, and results of structure refinement are summarized in Table 3 ▸. The hydrogen atoms of the sulfonamide NH_2_ and hydroxyl groups were localized in a difference-Fourier map and refined with O—H = 0.84 ± 0.01 Å, and with *U*_iso_(H) set to 1.5*U*_eq_(O) or 1.2*U*_eq_(N). All other hydrogen atoms were placed in calculated positions with C—H = 0.95 Å and refined using a riding model with fixed isotropic displacement parameters [*U*_iso_(H) = 1.2*U*_eq_(C)].

## Supplementary Material

Crystal structure: contains datablock(s) I. DOI: 10.1107/S2056989022002870/zn2017sup1.cif

Structure factors: contains datablock(s) I. DOI: 10.1107/S2056989022002870/zn2017Isup2.hkl

Supporting information file. DOI: 10.1107/S2056989022002870/zn2017Isup3.cml

CCDC reference: 2158517

Additional supporting information:  crystallographic information; 3D view; checkCIF report

## Figures and Tables

**Table 1:** Hydrogen-bond geometry (Å, °) *Cg*3, *Cg*4, *Cg*5 and *Cg*6 are the centroids of the C10*A*–C15*A*, N1*A*/C7*A*–C15*A*, N1*B*/C7*B*–C10*B*/C15*B* and C1*B*–C6*B* rings, respectively.

*D*—H⋯*A*	*D*—H	H⋯*A*	*D*⋯*A*	*D*—H⋯*A*
O1*B*—H1*B*⋯N1*B*	0.85 (2)	1.82 (3)	2.578 (3)	146 (4)
O1*A*—H1*A*⋯N1*A*	0.87 (2)	1.76 (3)	2.566 (4)	153 (6)
N2*B*—H2*BA*⋯O2*A* ^i^	0.87 (5)	2.20 (5)	2.878 (4)	135 (4)
N2*B*—H2*BB*⋯O3*A* ^ii^	0.87 (5)	2.13 (5)	2.908 (6)	149 (4)
N2*A*—H2*AA*⋯O3*B*	0.89 (5)	2.05 (5)	2.929 (6)	171 (5)
N2*A*—H2*AB*⋯O2*B* ^iii^	0.92 (5)	2.13 (5)	2.742 (5)	124 (4)
C13*B*—H13*B*⋯O2*B*	0.95	2.57	2.928 (4)	103
C8*B*—H8*B*⋯O1*B* ^iii^	0.95	2.76	3.191 (6)	109
C3*B*—H3*B*⋯O2*A* ^iv^	0.95	2.55	3.496 (4)	176
C14*B*—H14*B*⋯O1*B* ^v^	0.95	2.59	3.515 (4)	165
C14*A*—H14*A*⋯O1*A* ^vi^	0.95	2.48	3.419 (5)	170
C9*A*—H9*A*⋯*Cg*5^vii^	0.95	2.62	3.331 (3)	132
C9*B*—H9*B*⋯*Cg*4^i^	0.95	2.77	3.331 (5)	119
C9*B*—H9*B*⋯*Cg*3^i^	0.95	2.91	3.470 (5)	119
C5*A*—H5*A*⋯*Cg*6^vii^	0.95	2.89	3.566 (4)	129

Symmetry codes: (i) 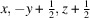 ; (ii) 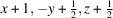 ; (iii) 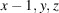 ; (iv) 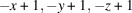 ; (v) 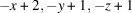 ; (vi) 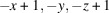 ; (vii) 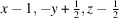 .

**Table 2:** Percentage contributions of inter­atomic contacts to the Hirshfeld surface

Contact	Percentage contribution
C⋯H/H⋯C	29.2
O⋯H/H⋯O	28.6
H⋯H	28.5
C⋯C	5.2
N⋯H/H⋯N	5
C⋯O/O⋯C	1.4
C⋯N/N⋯C	1.2
O⋯O	0.6
N⋯O/O⋯N	0.2
N⋯N	0.1

**Table 3:** Experimental details

Crystal data	
Chemical formula	C_15_H_12_N_2_O_3_S
*M*_r_	300.33
Crystal system, space group	Monoclinic, *P*2_1_/*c*
Temperature (K)	100
*a*, *b*, *c* (Å)	5.7667 (2), 28.4129 (7), 15.5339 (5)
β (°)	91.728 (3)
*V* (Å^3^)	2544.05 (14)
*Z*	8
Radiation type	Mo *K*α
μ (mm^−1^)	0.27
Crystal size (mm)	0.18 × 0.11 × 0.05

Data collection	
Diffractometer	Nonius KappaCCD
No. of measured, independent and observed [*I* > 2σ(*I*)] reflections	81539, 4473, 3058
*R*_int_	0.103
(sin θ/λ)_max_ (Å^−1^)	0.595

Refinement	
*R*[*F* ^2^ > 2σ(*F* ^2^)], *wR*(*F* ^2^), *S*	0.060, 0.184, 1.05
No. of reflections	4473
No. of parameters	397
No. of restraints	2
H-atom treatment	H atoms treated by a mixture of independent and constrained refinement
Δρ_max_, Δρ_min_ (e Å^−3^)	0.36, −0.58

Computer programs: *APEX2* and *SAINT* (Bruker, 2012 ▸); *SHELXT2018/2* (Sheldrick, 2015*a* ▸), *SHELXL2018/3* (Sheldrick, 2015*b* ▸), *OLEX2* (Dolomanov *et al.*, 2009 ▸) and *PLATON* (Spek, 2020 ▸).

## supplementary crystallographic information

### Crystal data

**Table:**

C_15_H_12_N_2_O_3_S	*F*(000) = 1248
*M**_r_* = 300.33	*D*_x_ = 1.568 Mg m^−^^3^
Monoclinic, *P*2_1_/*c*	Mo *K*α radiation, λ = 0.71073 Å
*a* = 5.7667 (2) Å	Cell parameters from 81539 reflections
*b* = 28.4129 (7) Å	θ = 3.4–25.0°
*c* = 15.5339 (5) Å	µ = 0.27 mm^−^^1^
β = 91.728 (3)°	*T* = 100 K
*V* = 2544.05 (14) Å^3^	Block, yellow
*Z* = 8	0.18 × 0.11 × 0.05 mm

### Data collection

**Table:**

Nonius KappaCCD diffractometer	*R*_int_ = 0.103
Radiation source: fine-focus sealed tube	θ_max_ = 25.0°, θ_min_ = 3.4°
ω scans	*h* = −6→6
81539 measured reflections	*k* = −33→33
4473 independent reflections	*l* = −18→18
3058 reflections with *I* > 2σ(*I*)	

### Refinement

**Table:**

Refinement on *F*^2^	2 restraints
Least-squares matrix: full	Hydrogen site location: mixed
*R*[*F*^2^ > 2σ(*F*^2^)] = 0.060	H atoms treated by a mixture of independent and constrained refinement
*wR*(*F*^2^) = 0.184	*w* = 1/[σ^2^(*F*_o_^2^) + (0.1002*P*)^2^ + 1.3957*P*] where *P* = (*F*_o_^2^ + 2*F*_c_^2^)/3
*S* = 1.05	(Δ/σ)_max_ < 0.001
4473 reflections	Δρ_max_ = 0.36 e Å^−^^3^
397 parameters	Δρ_min_ = −0.58 e Å^−^^3^

### Special details

**Table:**

Geometry. All esds (except the esd in the dihedral angle between two l.s. planes) are estimated using the full covariance matrix. The cell esds are taken into account individually in the estimation of esds in distances, angles and torsion angles; correlations between esds in cell parameters are only used when they are defined by crystal symmetry. An approximate (isotropic) treatment of cell esds is used for estimating esds involving l.s. planes.

### Fractional atomic coordinates and isotropic or equivalent isotropic displacement parameters (Å^2^)

**Table:**

	*x*	*y*	*z*	*U*_iso_*/*U*_eq_	
S1B	0.43707 (17)	0.26762 (3)	0.54873 (6)	0.0427 (3)	
S1A	0.0621 (2)	0.22144 (3)	0.29850 (7)	0.0542 (4)	
O1B	0.8560 (4)	0.54324 (8)	0.56908 (17)	0.0415 (6)	
H1B	0.793 (7)	0.5165 (9)	0.559 (3)	0.062*	
O2B	0.5936 (4)	0.25170 (9)	0.48546 (17)	0.0472 (7)	
O1A	0.3524 (4)	−0.04503 (9)	0.4612 (2)	0.0510 (7)	
O3B	0.1941 (5)	0.26115 (10)	0.5344 (2)	0.0608 (9)	
N1B	0.5920 (5)	0.47193 (9)	0.59665 (18)	0.0334 (7)	
O2A	0.3019 (6)	0.23513 (9)	0.29823 (19)	0.0604 (9)	
O3A	−0.0705 (6)	0.22426 (10)	0.22008 (19)	0.0656 (9)	
N1A	0.0991 (5)	0.02087 (10)	0.39571 (19)	0.0382 (7)	
N2B	0.5049 (8)	0.24069 (11)	0.6371 (2)	0.0552 (10)	
H2BA	0.380 (8)	0.2476 (17)	0.664 (3)	0.066*	
H2BB	0.653 (9)	0.2448 (17)	0.646 (3)	0.066*	
C15B	0.5513 (6)	0.42388 (11)	0.5876 (2)	0.0320 (8)	
C7B	0.4401 (5)	0.49941 (11)	0.6335 (2)	0.0328 (8)	
C10B	0.3518 (6)	0.40222 (11)	0.6190 (2)	0.0316 (8)	
C11B	0.3197 (6)	0.35382 (11)	0.6058 (2)	0.0316 (8)	
H11B	0.184184	0.338807	0.625647	0.038*	
C12B	0.4831 (6)	0.32827 (12)	0.5646 (2)	0.0323 (8)	
C7A	−0.0617 (6)	−0.00943 (12)	0.3720 (2)	0.0344 (8)	
C6B	0.4950 (6)	0.54968 (11)	0.6434 (2)	0.0318 (8)	
C1B	0.7007 (6)	0.56924 (12)	0.6119 (2)	0.0329 (8)	
C6A	−0.0177 (6)	−0.05960 (11)	0.3894 (2)	0.0332 (8)	
C5B	0.3413 (6)	0.57959 (12)	0.6855 (2)	0.0349 (8)	
H5B	0.201567	0.567044	0.706798	0.042*	
C13B	0.6843 (6)	0.34967 (12)	0.5342 (2)	0.0355 (8)	
H13B	0.797853	0.331376	0.506364	0.043*	
C14B	0.7158 (6)	0.39730 (12)	0.5450 (2)	0.0339 (8)	
H14B	0.849790	0.412131	0.523458	0.041*	
N2A	−0.0595 (10)	0.25402 (12)	0.3687 (3)	0.0700 (14)	
H2AA	0.030 (9)	0.258 (2)	0.416 (3)	0.084*	
H2AB	−0.215 (9)	0.2471 (19)	0.371 (3)	0.084*	
C1A	0.1874 (6)	−0.07528 (12)	0.4322 (2)	0.0369 (8)	
C2B	0.7453 (6)	0.61686 (12)	0.6239 (2)	0.0381 (8)	
H2B	0.884080	0.629988	0.602850	0.046*	
C3B	0.5928 (6)	0.64522 (12)	0.6654 (2)	0.0397 (9)	
H3B	0.626395	0.677708	0.673074	0.048*	
C15A	0.0715 (6)	0.06803 (12)	0.3764 (2)	0.0402 (9)	
C4B	0.3887 (6)	0.62652 (12)	0.6964 (2)	0.0394 (9)	
H4B	0.282487	0.646201	0.725088	0.047*	
C3A	0.0600 (6)	−0.15561 (12)	0.4198 (2)	0.0413 (9)	
H3A	0.085728	−0.188177	0.430152	0.050*	
C5A	−0.1792 (7)	−0.09350 (13)	0.3627 (2)	0.0420 (9)	
H5A	−0.317622	−0.083773	0.333166	0.050*	
C12A	0.0561 (7)	0.16215 (12)	0.3321 (2)	0.0418 (9)	
C14A	0.2468 (6)	0.09824 (12)	0.4045 (3)	0.0432 (9)	
H14A	0.372049	0.086394	0.439259	0.052*	
C2A	0.2235 (6)	−0.12309 (12)	0.4460 (3)	0.0418 (9)	
H2A	0.363317	−0.133402	0.473891	0.050*	
C13A	0.2416 (7)	0.14491 (13)	0.3828 (3)	0.0441 (9)	
H13A	0.362635	0.165385	0.402071	0.053*	
C4A	−0.1436 (7)	−0.14054 (13)	0.3781 (3)	0.0467 (10)	
H4A	−0.257925	−0.162833	0.360184	0.056*	
C11A	−0.1244 (7)	0.13377 (13)	0.3064 (3)	0.0496 (10)	
H11A	−0.251397	0.146160	0.273282	0.060*	
C10A	−0.1193 (7)	0.08580 (13)	0.3298 (3)	0.0472 (10)	
C8B	0.2383 (9)	0.47967 (18)	0.6658 (3)	0.0719 (14)	
H8B	0.128880	0.499032	0.693443	0.086*	
C9B	0.1984 (9)	0.4318 (2)	0.6572 (3)	0.0758 (14)	
H9B	0.059364	0.419024	0.678730	0.091*	
C8A	−0.2589 (9)	0.00637 (19)	0.3285 (3)	0.0768 (15)	
H8A	−0.376457	−0.015445	0.311468	0.092*	
C9A	−0.2877 (10)	0.05392 (19)	0.3092 (4)	0.0781 (15)	
H9A	−0.427266	0.064244	0.281171	0.094*	
H1A	0.299 (10)	−0.0174 (11)	0.448 (4)	0.117*	

### Atomic displacement parameters (Å^2^)

**Table:**

	*U*^11^	*U*^22^	*U*^33^	*U*^12^	*U*^13^	*U*^23^
S1B	0.0467 (6)	0.0297 (5)	0.0529 (6)	−0.0118 (4)	0.0204 (4)	−0.0139 (4)
S1A	0.0887 (9)	0.0228 (5)	0.0533 (7)	0.0071 (5)	0.0400 (6)	0.0043 (4)
O1B	0.0353 (13)	0.0271 (13)	0.0627 (17)	−0.0064 (11)	0.0140 (12)	−0.0010 (12)
O2B	0.0527 (16)	0.0352 (15)	0.0547 (17)	−0.0069 (12)	0.0198 (13)	−0.0160 (12)
O1A	0.0411 (15)	0.0271 (14)	0.084 (2)	−0.0025 (12)	−0.0145 (14)	0.0052 (13)
O3B	0.0464 (17)	0.0471 (17)	0.090 (2)	−0.0215 (13)	0.0276 (15)	−0.0315 (15)
N1B	0.0345 (15)	0.0240 (15)	0.0421 (17)	−0.0030 (12)	0.0061 (13)	0.0014 (12)
O2A	0.092 (2)	0.0278 (14)	0.0642 (19)	−0.0076 (14)	0.0472 (17)	−0.0009 (12)
O3A	0.103 (2)	0.0396 (17)	0.0556 (19)	0.0231 (16)	0.0297 (17)	0.0169 (13)
N1A	0.0396 (17)	0.0273 (16)	0.0475 (18)	−0.0027 (13)	−0.0012 (13)	0.0015 (13)
N2B	0.079 (3)	0.0261 (17)	0.062 (2)	−0.0065 (18)	0.033 (2)	−0.0044 (15)
C15B	0.0348 (18)	0.0239 (17)	0.0372 (19)	−0.0069 (14)	0.0014 (15)	−0.0002 (14)
C7B	0.0320 (18)	0.0282 (18)	0.039 (2)	−0.0035 (14)	0.0089 (15)	0.0015 (15)
C10B	0.0305 (18)	0.0278 (18)	0.0371 (19)	−0.0003 (14)	0.0072 (15)	−0.0017 (14)
C11B	0.0317 (18)	0.0296 (18)	0.0339 (18)	−0.0100 (14)	0.0048 (14)	0.0000 (14)
C12B	0.0367 (19)	0.0264 (18)	0.0340 (18)	−0.0050 (14)	0.0077 (15)	−0.0045 (14)
C7A	0.0365 (19)	0.0290 (19)	0.037 (2)	−0.0016 (15)	−0.0010 (15)	0.0036 (15)
C6B	0.0355 (18)	0.0259 (18)	0.0341 (19)	−0.0009 (14)	0.0039 (15)	0.0020 (14)
C1B	0.0325 (18)	0.0273 (18)	0.0390 (19)	0.0003 (14)	0.0030 (15)	0.0047 (14)
C6A	0.0390 (19)	0.0273 (18)	0.0334 (18)	−0.0021 (15)	0.0044 (15)	0.0003 (14)
C5B	0.0370 (19)	0.0286 (19)	0.039 (2)	−0.0018 (15)	0.0048 (15)	0.0024 (15)
C13B	0.0370 (19)	0.0307 (19)	0.039 (2)	−0.0028 (15)	0.0103 (16)	−0.0058 (15)
C14B	0.0319 (18)	0.0320 (19)	0.0383 (19)	−0.0083 (15)	0.0074 (15)	−0.0026 (15)
N2A	0.118 (4)	0.0254 (18)	0.070 (3)	0.011 (2)	0.057 (3)	0.0023 (18)
C1A	0.0335 (19)	0.0277 (19)	0.050 (2)	−0.0021 (15)	0.0046 (16)	0.0023 (16)
C2B	0.038 (2)	0.0295 (19)	0.047 (2)	−0.0072 (15)	0.0000 (16)	0.0081 (16)
C3B	0.050 (2)	0.0253 (18)	0.043 (2)	−0.0021 (16)	−0.0039 (17)	0.0016 (15)
C15A	0.050 (2)	0.0251 (18)	0.046 (2)	0.0015 (16)	0.0096 (18)	0.0027 (16)
C4B	0.050 (2)	0.0282 (19)	0.040 (2)	0.0059 (16)	0.0029 (17)	−0.0004 (15)
C3A	0.053 (2)	0.0242 (19)	0.047 (2)	−0.0025 (16)	0.0100 (18)	0.0008 (16)
C5A	0.047 (2)	0.033 (2)	0.045 (2)	−0.0085 (16)	−0.0067 (17)	0.0027 (16)
C12A	0.058 (2)	0.0223 (18)	0.046 (2)	0.0005 (17)	0.0193 (19)	−0.0013 (16)
C14A	0.043 (2)	0.0271 (19)	0.059 (2)	−0.0030 (16)	0.0023 (18)	0.0013 (17)
C2A	0.043 (2)	0.029 (2)	0.054 (2)	0.0017 (16)	0.0056 (18)	0.0036 (17)
C13A	0.048 (2)	0.0265 (19)	0.058 (2)	−0.0043 (16)	0.0100 (19)	−0.0034 (17)
C4A	0.054 (2)	0.029 (2)	0.057 (2)	−0.0132 (17)	−0.0004 (19)	−0.0020 (17)
C11A	0.062 (3)	0.030 (2)	0.057 (2)	0.0102 (19)	0.001 (2)	0.0028 (18)
C10A	0.050 (2)	0.031 (2)	0.061 (3)	−0.0032 (18)	−0.0034 (19)	−0.0040 (18)
C8B	0.075 (3)	0.062 (3)	0.079 (4)	0.002 (3)	0.017 (3)	−0.004 (3)
C9B	0.074 (3)	0.075 (4)	0.079 (4)	−0.014 (3)	0.015 (3)	0.003 (3)
C8A	0.080 (4)	0.067 (3)	0.083 (4)	−0.011 (3)	0.000 (3)	−0.003 (3)
C9A	0.082 (3)	0.074 (4)	0.078 (4)	0.008 (3)	−0.009 (3)	0.003 (3)

### Geometric parameters (Å, º)

**Table:**

S1B—O2B	1.428 (2)	C5B—C4B	1.371 (5)
S1B—O3B	1.424 (3)	C13B—H13B	0.9500
S1B—N2B	1.610 (4)	C13B—C14B	1.375 (5)
S1B—C12B	1.760 (3)	C14B—H14B	0.9500
S1A—O2A	1.437 (3)	N2A—H2AA	0.89 (5)
S1A—O3A	1.421 (4)	N2A—H2AB	0.92 (5)
S1A—N2A	1.607 (4)	C1A—C2A	1.390 (5)
S1A—C12A	1.764 (4)	C2B—H2B	0.9500
O1B—H1B	0.854 (19)	C2B—C3B	1.369 (5)
O1B—C1B	1.351 (4)	C3B—H3B	0.9500
O1A—C1A	1.350 (4)	C3B—C4B	1.390 (5)
O1A—H1A	0.87 (2)	C15A—C14A	1.387 (5)
N1B—C15B	1.392 (4)	C15A—C10A	1.393 (5)
N1B—C7B	1.317 (4)	C4B—H4B	0.9500
N1A—C7A	1.310 (4)	C3A—H3A	0.9500
N1A—C15A	1.381 (4)	C3A—C2A	1.373 (5)
N2B—H2BA	0.87 (5)	C3A—C4A	1.392 (5)
N2B—H2BB	0.87 (5)	C5A—H5A	0.9500
C15B—C10B	1.405 (4)	C5A—C4A	1.372 (5)
C15B—C14B	1.394 (5)	C12A—C13A	1.398 (5)
C7B—C6B	1.470 (5)	C12A—C11A	1.367 (5)
C7B—C8B	1.398 (6)	C14A—H14A	0.9500
C10B—C11B	1.401 (5)	C14A—C13A	1.368 (5)
C10B—C9B	1.369 (6)	C2A—H2A	0.9500
C11B—H11B	0.9500	C13A—H13A	0.9500
C11B—C12B	1.365 (4)	C4A—H4A	0.9500
C12B—C13B	1.404 (4)	C11A—H11A	0.9500
C7A—C6A	1.472 (5)	C11A—C10A	1.411 (5)
C7A—C8A	1.380 (6)	C10A—C9A	1.359 (6)
C6B—C1B	1.411 (5)	C8B—H8B	0.9500
C6B—C5B	1.403 (5)	C8B—C9B	1.385 (7)
C1B—C2B	1.389 (5)	C9B—H9B	0.9500
C6A—C1A	1.411 (5)	C8A—H8A	0.9500
C6A—C5A	1.394 (5)	C8A—C9A	1.392 (7)
C5B—H5B	0.9500	C9A—H9A	0.9500
			
O2B—S1B—N2B	107.10 (19)	S1A—N2A—H2AB	110 (3)
O2B—S1B—C12B	108.09 (15)	H2AA—N2A—H2AB	122 (5)
O3B—S1B—O2B	119.39 (17)	O1A—C1A—C6A	121.9 (3)
O3B—S1B—N2B	106.6 (2)	O1A—C1A—C2A	118.0 (3)
O3B—S1B—C12B	106.96 (16)	C2A—C1A—C6A	120.0 (3)
N2B—S1B—C12B	108.33 (17)	C1B—C2B—H2B	119.4
O2A—S1A—N2A	106.6 (2)	C3B—C2B—C1B	121.2 (3)
O2A—S1A—C12A	106.65 (18)	C3B—C2B—H2B	119.4
O3A—S1A—O2A	118.37 (18)	C2B—C3B—H3B	120.0
O3A—S1A—N2A	108.3 (2)	C2B—C3B—C4B	120.1 (3)
O3A—S1A—C12A	107.01 (19)	C4B—C3B—H3B	120.0
N2A—S1A—C12A	109.67 (18)	N1A—C15A—C14A	117.0 (3)
C1B—O1B—H1B	107 (3)	N1A—C15A—C10A	123.3 (3)
C1A—O1A—H1A	105 (4)	C14A—C15A—C10A	119.7 (3)
C7B—N1B—C15B	120.9 (3)	C5B—C4B—C3B	119.7 (3)
C7A—N1A—C15A	120.0 (3)	C5B—C4B—H4B	120.1
S1B—N2B—H2BA	97 (3)	C3B—C4B—H4B	120.1
S1B—N2B—H2BB	106 (3)	C2A—C3A—H3A	120.2
H2BA—N2B—H2BB	136 (4)	C2A—C3A—C4A	119.5 (3)
N1B—C15B—C10B	122.1 (3)	C4A—C3A—H3A	120.2
N1B—C15B—C14B	117.7 (3)	C6A—C5A—H5A	119.1
C14B—C15B—C10B	120.2 (3)	C4A—C5A—C6A	121.7 (4)
N1B—C7B—C6B	118.5 (3)	C4A—C5A—H5A	119.1
N1B—C7B—C8B	119.3 (3)	C13A—C12A—S1A	118.7 (3)
C8B—C7B—C6B	122.1 (3)	C11A—C12A—S1A	119.9 (3)
C11B—C10B—C15B	119.0 (3)	C11A—C12A—C13A	121.4 (3)
C9B—C10B—C15B	115.4 (3)	C15A—C14A—H14A	119.6
C9B—C10B—C11B	125.5 (3)	C13A—C14A—C15A	120.8 (4)
C10B—C11B—H11B	120.0	C13A—C14A—H14A	119.6
C12B—C11B—C10B	120.0 (3)	C1A—C2A—H2A	119.5
C12B—C11B—H11B	120.0	C3A—C2A—C1A	120.9 (3)
C11B—C12B—S1B	118.9 (2)	C3A—C2A—H2A	119.5
C11B—C12B—C13B	121.1 (3)	C12A—C13A—H13A	120.4
C13B—C12B—S1B	120.0 (2)	C14A—C13A—C12A	119.3 (3)
N1A—C7A—C6A	117.9 (3)	C14A—C13A—H13A	120.4
N1A—C7A—C8A	119.3 (4)	C3A—C4A—H4A	120.0
C8A—C7A—C6A	122.7 (4)	C5A—C4A—C3A	120.0 (3)
C1B—C6B—C7B	121.8 (3)	C5A—C4A—H4A	120.0
C5B—C6B—C7B	120.0 (3)	C12A—C11A—H11A	120.5
C5B—C6B—C1B	118.2 (3)	C12A—C11A—C10A	119.0 (4)
O1B—C1B—C6B	122.1 (3)	C10A—C11A—H11A	120.5
O1B—C1B—C2B	118.4 (3)	C15A—C10A—C11A	119.6 (4)
C2B—C1B—C6B	119.4 (3)	C9A—C10A—C15A	115.4 (4)
C1A—C6A—C7A	122.0 (3)	C9A—C10A—C11A	125.0 (4)
C5A—C6A—C7A	120.3 (3)	C7B—C8B—H8B	120.1
C5A—C6A—C1A	117.7 (3)	C9B—C8B—C7B	119.8 (4)
C6B—C5B—H5B	119.3	C9B—C8B—H8B	120.1
C4B—C5B—C6B	121.4 (3)	C10B—C9B—C8B	122.5 (4)
C4B—C5B—H5B	119.3	C10B—C9B—H9B	118.8
C12B—C13B—H13B	120.3	C8B—C9B—H9B	118.8
C14B—C13B—C12B	119.5 (3)	C7A—C8A—H8A	119.6
C14B—C13B—H13B	120.3	C7A—C8A—C9A	120.8 (5)
C15B—C14B—H14B	119.9	C9A—C8A—H8A	119.6
C13B—C14B—C15B	120.1 (3)	C10A—C9A—C8A	121.1 (5)
C13B—C14B—H14B	119.9	C10A—C9A—H9A	119.5
S1A—N2A—H2AA	112 (4)	C8A—C9A—H9A	119.5
			
S1B—C12B—C13B—C14B	178.4 (3)	C7A—N1A—C15A—C14A	179.1 (3)
S1A—C12A—C13A—C14A	−175.7 (3)	C7A—N1A—C15A—C10A	−1.9 (5)
S1A—C12A—C11A—C10A	176.4 (3)	C7A—C6A—C1A—O1A	1.4 (5)
O1B—C1B—C2B—C3B	178.9 (3)	C7A—C6A—C1A—C2A	−178.9 (3)
O2B—S1B—C12B—C11B	164.4 (3)	C7A—C6A—C5A—C4A	−179.9 (3)
O2B—S1B—C12B—C13B	−15.0 (3)	C7A—C8A—C9A—C10A	2.4 (8)
O1A—C1A—C2A—C3A	178.5 (3)	C6B—C7B—C8B—C9B	−177.9 (4)
O3B—S1B—C12B—C11B	34.7 (3)	C6B—C1B—C2B—C3B	−0.2 (5)
O3B—S1B—C12B—C13B	−144.7 (3)	C6B—C5B—C4B—C3B	0.0 (5)
N1B—C15B—C10B—C11B	178.6 (3)	C1B—C6B—C5B—C4B	−0.3 (5)
N1B—C15B—C10B—C9B	1.4 (5)	C1B—C2B—C3B—C4B	−0.1 (5)
N1B—C15B—C14B—C13B	−180.0 (3)	C6A—C7A—C8A—C9A	−176.4 (4)
N1B—C7B—C6B—C1B	2.3 (5)	C6A—C1A—C2A—C3A	−1.2 (5)
N1B—C7B—C6B—C5B	−177.4 (3)	C6A—C5A—C4A—C3A	−1.2 (6)
N1B—C7B—C8B—C9B	−1.2 (7)	C5B—C6B—C1B—O1B	−178.6 (3)
O2A—S1A—C12A—C13A	25.1 (3)	C5B—C6B—C1B—C2B	0.4 (5)
O2A—S1A—C12A—C11A	−153.1 (3)	C14B—C15B—C10B—C11B	−0.8 (5)
O3A—S1A—C12A—C13A	152.7 (3)	C14B—C15B—C10B—C9B	−178.0 (4)
O3A—S1A—C12A—C11A	−25.5 (4)	N2A—S1A—C12A—C13A	−90.0 (4)
N1A—C7A—C6A—C1A	1.7 (5)	N2A—S1A—C12A—C11A	91.8 (4)
N1A—C7A—C6A—C5A	−177.7 (3)	C1A—C6A—C5A—C4A	0.8 (5)
N1A—C7A—C8A—C9A	0.4 (7)	C2B—C3B—C4B—C5B	0.2 (5)
N1A—C15A—C14A—C13A	175.2 (3)	C15A—N1A—C7A—C6A	176.3 (3)
N1A—C15A—C10A—C11A	−174.5 (3)	C15A—N1A—C7A—C8A	−0.6 (6)
N1A—C15A—C10A—C9A	4.5 (6)	C15A—C14A—C13A—C12A	0.3 (6)
N2B—S1B—C12B—C11B	−79.9 (3)	C15A—C10A—C9A—C8A	−4.6 (7)
N2B—S1B—C12B—C13B	100.7 (3)	C5A—C6A—C1A—O1A	−179.3 (3)
C15B—N1B—C7B—C6B	178.4 (3)	C5A—C6A—C1A—C2A	0.4 (5)
C15B—N1B—C7B—C8B	1.6 (5)	C12A—C11A—C10A—C15A	−1.7 (6)
C15B—C10B—C11B—C12B	1.3 (5)	C12A—C11A—C10A—C9A	179.4 (4)
C15B—C10B—C9B—C8B	−1.0 (7)	C14A—C15A—C10A—C11A	4.5 (6)
C7B—N1B—C15B—C10B	−1.8 (5)	C14A—C15A—C10A—C9A	−176.5 (4)
C7B—N1B—C15B—C14B	177.6 (3)	C2A—C3A—C4A—C5A	0.5 (6)
C7B—C6B—C1B—O1B	1.7 (5)	C13A—C12A—C11A—C10A	−1.8 (6)
C7B—C6B—C1B—C2B	−179.3 (3)	C4A—C3A—C2A—C1A	0.7 (6)
C7B—C6B—C5B—C4B	179.4 (3)	C11A—C12A—C13A—C14A	2.5 (6)
C7B—C8B—C9B—C10B	0.9 (8)	C11A—C10A—C9A—C8A	174.3 (5)
C10B—C15B—C14B—C13B	−0.5 (5)	C10A—C15A—C14A—C13A	−3.8 (6)
C10B—C11B—C12B—S1B	−179.8 (3)	C8B—C7B—C6B—C1B	179.0 (4)
C10B—C11B—C12B—C13B	−0.4 (5)	C8B—C7B—C6B—C5B	−0.7 (5)
C11B—C10B—C9B—C8B	−178.0 (4)	C9B—C10B—C11B—C12B	178.1 (4)
C11B—C12B—C13B—C14B	−1.0 (5)	C8A—C7A—C6A—C1A	178.5 (4)
C12B—C13B—C14B—C15B	1.4 (5)	C8A—C7A—C6A—C5A	−0.8 (6)

### Hydrogen-bond geometry (Å, º)

*Cg*3, *Cg*4, *Cg*5 and *Cg*6 are the centroids of the C10*A*–C15*A*, N1*A*/C7*A*–C15*A*, N1*B*/C7*B*–C10*B*/C15*B* and C1*B*–C6*B* rings, respectively.

**Table:**

*D*—H···*A*	*D*—H	H···*A*	*D*···*A*	*D*—H···*A*
O1*B*—H1*B*···N1*B*	0.85 (2)	1.82 (3)	2.578 (3)	146 (4)
O1*A*—H1*A*···N1*A*	0.87 (2)	1.76 (3)	2.566 (4)	153 (6)
N2*B*—H2*BA*···O2*A*^i^	0.87 (5)	2.20 (5)	2.878 (4)	135 (4)
N2*B*—H2*BB*···O3*A*^ii^	0.87 (5)	2.13 (5)	2.908 (6)	149 (4)
N2*A*—H2*AA*···O3*B*	0.89 (5)	2.05 (5)	2.929 (6)	171 (5)
N2*A*—H2*AB*···O2*B*^iii^	0.92 (5)	2.13 (5)	2.742 (5)	124 (4)
C13*B*—H13*B*···O2*B*	0.95	2.57	2.928 (4)	103
C8*B*—H8*B*···O1*B*^iii^	0.95	2.76	3.191 (6)	109
C3*B*—H3*B*···O2*A*^iv^	0.95	2.55	3.496 (4)	176
C14*B*—H14*B*···O1*B*^v^	0.95	2.59	3.515 (4)	165
C14*A*—H14*A*···O1*A*^vi^	0.95	2.48	3.419 (5)	170
C9*A*—H9*A*···*Cg*5^vii^	0.95	2.62	3.331 (3)	132
C9*B*—H9*B*···*Cg*4^i^	0.95	2.77	3.331 (5)	119
C9*B*—H9*B*···*Cg*3^i^	0.95	2.91	3.470 (5)	119
C5*A*—H5*A*···*Cg*6^vii^	0.95	2.89	3.566 (4)	129

Symmetry codes: (i) *x*, −*y*+1/2, *z*+1/2; (ii) *x*+1, −*y*+1/2, *z*+1/2; (iii) *x*−1, *y*, *z*; (iv) −*x*+1, −*y*+1, −*z*+1; (v) −*x*+2, −*y*+1, −*z*+1; (vi) −*x*+1, −*y*, −*z*+1; (vii) *x*−1, −*y*+1/2, *z*−1/2.

## References

[bb1] Alexandre, P.-E., Zhang, W.-S., Rominger, F., Elbert, S. M., Schröder, R. R. & Mastalerz, M. (2020). *Angew. Chem. Int. Ed.* **59**, 19675–19679. 10.1002/anie.202007048 PMC7689861 32521080

[bb2] Al-Shabib, N. A., Khan, J. M., Malik, A., Rehman, M. T., AlAjmi, M. F., Husain, F. M., Ahmed, M. Z. & Alamery, S. F. (2020). *J. Mol. Liq.* **311**, 113215–113221.

[bb31] Bruker (2012). *APEX2* and *SAINT*. Bruker AXS Inc, Madison, Wisconsin, USA.

[bb3] Chi, N. T. T., Thong, P. V., Mai, T. T. C. & Van Meervelt, L. (2018). *Acta Cryst.* C**74**, 1732–1743. 10.1107/S2053229618015978 30516159

[bb4] Dolomanov, O. V., Bourhis, L. J., Gildea, R. J., Howard, J. A. K. & Puschmann, H. (2009). *J. Appl. Cryst.* **42**, 339–341.

[bb5] Elbert, S. M., Wagner, P., Kanagasundaram, T., Rominger, F. & Mastalerz, M. (2017). *Chem. Eur. J.* **23**, 935–945. 10.1002/chem.201604421 27862420

[bb6] Elgawad, H. A., Alhusseiny, S. M., Taman, A., Youssef, M. Y., Mansour, B., Massoud, M. & Handousa, A. (2019). *Exp. Parasitol.* **206**, 107756–107765. 10.1016/j.exppara.2019.107756 31494217

[bb7] Ferreira, J. P. S., Cardoso, S. M., Almeida Paz, F. A., Silva, A. M. S. & Silva, V. L. M. (2020). *New J. Chem.* **44**, 6501–6509.

[bb8] Galai, M., Rbaa, M., Ouakki, M., Dahmani, K., Kaya, S., Arrousse, N., Dkhireche, N., Briche, S., Lakhrissi, B. & Ebn Touhami, M. (2021). *Chem. Phys. Lett.* **776**, 138700–138720.

[bb9] Goel, S., Yadav, H., Sinha, N., Singh, B., Bdikin, I. & Kumar, B. (2018). *Acta Cryst.* B**74**, 12–23. 10.1107/S2052520617002906 28572545

[bb10] Groom, C. R., Bruno, I. J., Lightfoot, M. P. & Ward, S. C. (2016). *Acta Cryst.* B**72**, 171–179. 10.1107/S2052520616003954 PMC4822653 27048719

[bb11] Guo, Q.-S., Lu, Y.-N., Liu, B., Xiao, J. & Li, J.-S. (2006). *J. Organomet. Chem.* **691**, 1282–1287.

[bb12] Han, Y.-P., Li, X.-S., Sun, Z., Zhu, X.-Y., Li, M., Song, X.-R. & Liang, Y.-M. (2017). *Adv. Synth. Catal.* **359**, 2735–2740.

[bb13] Hassan, K. M., Shaban, E., Elhaddad, G. M., Shokair, S. H., Pannipara, M. & ElSayed, I. E. (2022). *J. King Saud Univ. Sci.* **34**, 101670–101678.

[bb14] Lavanya, G., Magesh, C. J., Venkatapathy, K., Perumal, P. T. & Prema, S. (2021). *Bioorg. Chem.* **107**, 104582–104596. 10.1016/j.bioorg.2020.104582 33450547

[bb15] Mague, J. T., Mohamed, S. K., Akkurt, M., Albayati, M. R. & Ahmed, E. A. (2016). *IUCrData*, **1**, x161544–x161546.

[bb16] McKinnon, J. J., Jayatilaka, D. & Spackman, M. A. (2007). *Chem. Commun.* 3814–3816. 10.1039/b704980c 18217656

[bb17] Mulakayala, N., Rambabu, D., Raja, M. R. M. C., Kumar, C. S., Kalle, A. M., Rama Krishna, G., Malla Reddy, C., Basaveswara Rao, M. V. & Pal, M. (2012). *Bioorg. Med. Chem.* **20**, 759–768. 10.1016/j.bmc.2011.12.001 22202437

[bb18] Redshaw, C. & Tang, Y. (2012). *Chem. Soc. Rev.* **41**, 4484–4510. 10.1039/c2cs35028a 22592513

[bb19] Sheldrick, G. M. (2015*a*). *Acta Cryst.* A**71**, 3–8.

[bb32] Sheldrick, G. M. (2015*b*). *Acta Cryst.* C**71**, 3–8.

[bb20] Shishkina, S. V., Levandovskiy, I. A., Ukrainets, I. V., Sidorenko, L. V., Grinevich, L. A. & Yanchuk, I. B. (2018). *Acta Cryst.* C**74**, 1759–1767. 10.1107/S2053229618016352 30516162

[bb21] Shrungesh Kumar, T. O., Naveen, S., Kumara, M. N., Mahadevan, K. M. & Lokanath, N. K. (2015). *Acta Cryst.* E**71**, o514–o515. 10.1107/S2056989015011706 PMC4518944 26279938

[bb22] Spackman, M. A. & Jayatilaka, D. (2009). *CrystEngComm*, **11**, 19–32.

[bb23] Spackman, P. R., Turner, M. J., McKinnon, J. J., Wolff, S. K., Grimwood, D. J., Jayatilaka, D. & Spackman, M. A. (2021). *J. Appl. Cryst.* **54**, 1006–1011. 10.1107/S1600576721002910 PMC8202033 34188619

[bb24] Spek, A. L. (2020). *Acta Cryst.* E**76**, 1–11. 10.1107/S2056989019016244 PMC6944088 31921444

[bb25] Twaróg, K., Hołyńska, M. & Kochel, A. (2020). *Acta Cryst.* C**76**, 500–506. 10.1107/S2053229620004593 32367832

[bb26] Wu, X.-W., Wu, W.-F., Yin, S. & Ma, J.-P. (2015). *Acta Cryst.* C**71**, 683–689. 10.1107/S2053229615012929 26243415

[bb27] Yadav, P. & Shah, K. (2021). *Bioorg. Chem.* **109**, 104639–104680. 10.1016/j.bioorg.2021.104639 33618829

[bb28] Yao, C., Qin, B., Zhang, H., Lu, J., Wang, D. & Tu, S. (2012). *RSC Adv.* **2**, 3759–3764.

[bb29] Yu, H., Yang, J.-X., Han, J.-Q., Li, P.-F., Hou, Y.-L., Wang, W.-M. & Fang, M. (2019). *New J. Chem.* **43**, 8067–8074.

[bb30] Zheng, Y.-H., Lu, H.-Y., Li, M. & Chen, C.-F. (2013). *Eur. J. Org. Chem.* pp. 3059–3066.

